# Recovery of Distal Arm Movements in Spinal Cord Injured Patients with a Body-Machine Interface: A Proof-of-Concept Study

**DOI:** 10.3390/s21062243

**Published:** 2021-03-23

**Authors:** Camilla Pierella, Elisa Galofaro, Alice De Luca, Luca Losio, Simona Gamba, Antonino Massone, Ferdinando A. Mussa-Ivaldi, Maura Casadio

**Affiliations:** 1 Department of Neuroscience, Rehabilitation, Ophthalmology, Genetics, Maternal and Child Health (DINOGMI), University of Genova, 16132 Genoa, Italy; 2 Department of Informatics, Bioengineering, Robotics and Systems Engineering (DIBRIS), University of Genoa, 16145 Genoa, Italy; elisa.galofaro@edu.unige.it (E.G.); alice.deluca@movendo.technology (A.D.L.); 3 Department of Physiology, Northwestern University, Chicago, IL 60611, USA; sandro@northwestern.edu; 4 Shirley Ryan Ability Lab, Chicago, IL 60611, USA; 5 Assistive Robotics and Interactive Exosuits (ARIES) Lab, Institute of Computer Engineering (ZITI), University of Heidelberg, 69117 Heidelberg, Germany; 6 Movendo Technology, 16128 Genoa, Italy; 7 Recovery and Functional Reeducation Unit, Santa Corona Hospital, ASL2 Savonese, 17027 Pietra Ligure, Italy; 8 S.C. Unità Spinale Unipolare, Santa Corona Hospital, ASL2 Savonese, 17027 Pietra Ligure, Italy; l.losio@asl2.liguria.it (L.L.); s.gamba@asl2.liguria.it (S.G.); a.massone@asl2.liguria.it (A.M.); 9 Italian Spinal Cord Laboratory (SCIL), 17027 Pietra Ligure, Italy; 10 Department of Physical Medicine and Rehabilitation, Northwestern University, Evanston, IL 60208, USA; 11 Department of Biomedical Engineering, Northwestern University, Evanston, IL 60208, USA

**Keywords:** motor learning, neurorehabilitation, body-machine interface, spinal cord injury, motion tracking

## Abstract

Background: The recovery of upper limb mobility and functions is essential for people with cervical spinal cord injuries (cSCI) to maximize independence in daily activities and ensure a successful return to normality. The rehabilitative path should include a thorough neuromotor evaluation and personalized treatments aimed at recovering motor functions. Body-machine interfaces (BoMI) have been proven to be capable of harnessing residual joint motions to control objects like computer cursors and virtual or physical wheelchairs and to promote motor recovery. However, their therapeutic application has still been limited to shoulder movements. Here, we expanded the use of BoMI to promote the whole arm’s mobility, with a special focus on elbow movements. We also developed an instrumented evaluation test and a set of kinematic indicators for assessing residual abilities and recovery. Methods: Five inpatient cSCI subjects (four acute, one chronic) participated in a BoMI treatment complementary to their standard rehabilitative routine. The subjects wore a BoMI with sensors placed on both proximal and distal arm districts and practiced for 5 weeks. The BoMI was programmed to promote symmetry between right and left arms use and the forearms’ mobility while playing games. To evaluate the effectiveness of the treatment, the subjects’ kinematics were recorded while performing an evaluation test that involved functional bilateral arms movements, before, at the end, and three months after training. Results: At the end of the training, all subjects learned to efficiently use the interface despite being compelled by it to engage their most impaired movements. The subjects completed the training with bilateral symmetry in body recruitment, already present at the end of the familiarization, and they increased the forearm activity. The instrumental evaluation confirmed this. The elbow motion’s angular amplitude improved for all subjects, and other kinematic parameters showed a trend towards the normality range. Conclusion: The outcomes are preliminary evidence supporting the efficacy of the proposed BoMI as a rehabilitation tool to be considered for clinical practice. It also suggests an instrumental evaluation protocol and a set of indicators to assess and evaluate motor impairment and recovery in cSCI.

## 1. Introduction

Spinal Cord Injury (SCI) results in deep and devastating life changes connected to the loss of motor and/or sensory functions below the level of the lesion [1,2]. This condition is not only physically but also psychologically challenging for SCI people because one of the main and impacting consequences is the loss of functional independence in activities of daily living (ADLs), making recovery a priority for SCI individuals [2,3]. To this aim, physical rehabilitation continues to remain a mainstay in the treatment of SCI because, so far, no curative treatments exist and only limited spontaneous recovery attributed to the natural and intrinsic neural plasticity of the remaining intact fibers happens after the lesion occurrence [2,3,4]. Commonly, the rehabilitation treatments, administered manually by the therapist, depend on the injury level and can follow diversified approaches based on each individual’s needs [5,6,7]. They are not fully resolutive and are focused on maximizing residual motor skills or overcoming inabilities by teaching compensatory strategies or using assistive devices [4,6,8].

In the last decade, robotic devices and other technologies have been integrated into training programs of people with neuromotor disabilities, like SCI, with promising results [9,10,11]. In parallel with the development of robots and their introduction into clinical practice, there has also been fast progress in advancing body-machine interfaces (BoMIs). Such interfaces transform user’ body movements to control signals for external assistive devices [12,13,14], and have been considered as a safe means for achieving rehabilitative goals [15,16]. A BoMI normally requires the application of surface sensors to different parts of the body that the user is still capable to move or that need to be treated in a therapeutic intervention. The general BoMI goal is to allow the user to control, through active movements, external devices such as personal computers, wheelchairs, and assistive manipulators. Typically, the BoMI exploits two key features of the motor control system: redundancy and plasticity. Redundancy, as suggested by Bernstein [17], can be employed by the BoMIs in two manners: (a) to explore an overabundant number of body signals for extracting the best signal subspace, and (b) to find new natural subsets of solutions, either when the users’ ability decreases for the progression of a related pathology, or as it increases as a consequence of the treatment or of motor learning. Neural plasticity refers to the reorganization of connections within the central nervous system allowing the assignment of new functions to the available capacity for movement’s control. The exploitation of redundancy also requires a reorganization, or remapping, of the residual ability to control body motions. When subjects use movements of the eye [18,19], head [20,21], shoulders [22,23,24], or tongue [25,26] for driving a wheelchair or piloting a robotic arm, they associate controlling these parts of the body with functions that before the injury were performed by other parts.

Earlier studies tested the efficacy of a new generation of BoMI harnessing the spared abilities of the upper body after a cervical spinal cord injury (cSCI) [15,23,27,28]. In particular, such studies exploit small shoulder residual movements to control a power wheelchair [23], or low-cost BoMI in which patients can receive personalized therapy, even within the home environment, with sensors placed exclusively on the shoulders and arms [15,27,28].Depending on the severity of injury, survivors of spinal cord injury, retain some movement, which can be used to control assistive devices such as power wheelchairs or a computer. These studies, mapping shoulder movements onto control signals for computers or virtual and power wheelchairs, demonstrated that a BoMI based on inertial measurement units (IMUs) capturing natural movements facilitates the exploration of new motor patterns by recognizing silent or weak abilities and targeting them with specific exercises. The combination of assistive and rehabilitative functions [15,16,29] could result in complementary benefits for the BoMI users: the motor skills that are partially recovered through training can be reprocessed and included in the control of the same interface, making it continuously customized and calibrated on the evolving abilities of its user.

In this study, the BoMI sensors were placed on both arms and forearms, as opposed to shoulders and arms like in previous studies, with the rationale that more distal limb regions are more challenging for people with cSCI to control. The objective was to do a retrospective study on the efficacy of the BoMI in the clinical practice as an instrument for physical therapists (PTs) to lead high-level cSCI subjects toward their rehabilitative goals.

In particular, we aimed at quantifying: (i) the effects of using the BoMI to recover distal body movements in cSCI patients; (ii) the possible motor changes that occurred after the training with the BoMI; (iii) the effects of the BoMI-supported training with an analysis of the cSCI subjects’ movement recorded at the beginning, at the end, and one month after the end of the training. Our results shown that with the current BoMI, we were able to tailor personalized rehabilitative interventions increasing the use of the distal parts of the upper body and motivating the subjects to explore a larger range of movement.

## 2. Materials and Methods

### 2.1. Experimental Setup and Protocol

**Figure 1:**
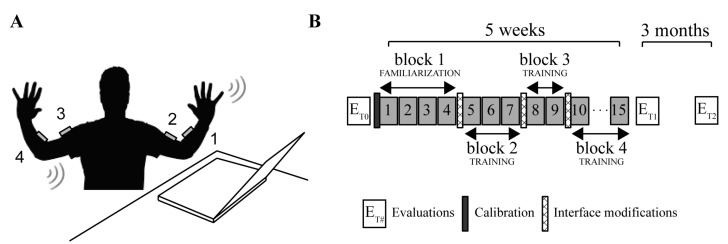
Experimental setup and protocol. (**A**) The subject sits in front of a computer wearing four IMUs that communicate wireless with the computer. With upper body movements the user is controlling the movements of a virtual cursor. (**B**) The subject had evaluation sessions before (ET0), at the end (ET1) and three months after the end of the training with the BoMI (ET2). The practice with the BoMI consisted of 15 sessions with increasing difficulty, which can be grouped into four main blocks (block1: familiarization and blocks 2–4: training blocks), in which subjects performed a set of different tasks.

In this study, we used four wireless and low-cost inertial measurement units (IMUs) (Yei Technology, 3-Space Sensor™ Wireless) placed on a garment attached by Velcro™ strips to the upper arms and forearms. The sensors were positioned on the distal portions of the upper body as shown in Figure 1A: sensor 1 on the left forearm, sensor 2 on the left arm, sensor 3 on the right arm and sensor 4 on the right forearm.

Each IMU, combining the information of a triaxial gyroscope, accelerometer and compass sensors embedded in the IMU, in conjunction with on-board filtering algorithms, provided in real-time pitch and roll angles. Thus, the system generated, at every instant n, an 8-dimensional signal vector $q^{\left(n\right)}=\left[q_{1}{}^{\left(n\right)},q_{2}{}^{\left(n\right)},\;\cdots ,q_{8}{}^{\left(n\right)}\right]{\;}^{T}$ including the output (pitch, roll) of all sensors. During the calibration phase, at the beginning of the first session, each subject was instructed to perform self-directed and self-paced upper-body motions—described as a free body-dance—for 1 min. In this phase, the IMU’s signals were continuously recorded. Then, through principal component analysis (PCA), we identified the plane of maximum body mobility embedded within the space of the IMU signals, taking the first two eigenvectors, $h_{1}=\left[h_{1,1},h_{1,2},\;\cdots ,h_{1,8}\right]{\;}^{T}$ and $h_{2}=\left[h_{2,1},h_{2,2},\;\cdots ,h_{2,8}\right]{\;}^{T}$, of the covariance matrix and combining them in a matrix H that generated the linear mapping from body to cursor vectors:

(1) $$p^{\left(n\right)}=\left[\begin{array}{c} \begin{array}{ccc} h_{1,1} & \cdots & h_{1,8} \end{array}\\ \begin{array}{ccc} h_{2,1} & \cdots & h_{2,8} \end{array} \end{array}\right]\cdot q^{\left(n\right)}=H\cdot q^{\left(n\right)}$$

where $q^{\left(n\right)}$ is the 8-dimensional “body vector” and $p^{\left(n\right)}$ the 2-dimensional control vector encoding the position of a computer cursor. More details of this procedure are in [30]. By establishing a correspondence of the task space—the space of cursor’s positions—with this plane, we associated the control variables with the degrees of freedom that the impaired subjects spontaneously used with the greatest ease.

The training protocol (Figure 1B) consisted of 15 sessions of practice with the BoMI of about 45 min that were repeated for 5 weeks. The sessions were organized in four main blocks of increasing difficulty: 1 block of familiarization and 3 blocks of training. Through these, the user completed a series of different tasks, already adopted in [15,16]:

- Reaching. The subjects, starting from the center of the screen, had to reach for three times eight external targets equally spaced in eight directions (0°, 45°, 90°, 135°, 180°, 225°, 270°, 315°), for a total of 24 center-out reaching movements. The external target was positioned at 8.5 cm from the center and appeared randomly in each of the eight directions. The subjects were to reach the external target before it changed color from green to red and then to come back to the central one. This color change happened 1 s after the external target appeared. The target was considered acquired when the cursor remained inside it for 500 ms. The reaching task was performed at the beginning (named 1st Reaching) and at the end (named 2nd Reaching) of each session.
- Vertical pong simulation. The subjects, by controlling the x and y coordinate of a paddle, were asked to hit a ball moving in the 2-d space of the game field. The prevalent motion of the ball was along the vertical direction (up/down). Subjects obtained a point for every hit, sending the ball to bounce off the top wall. During each session subjects played five epochs of pong, each lasting 2.5 min.
- Horizontal pong simulation. This task was the same as the vertical pong, but the movement of the ball was mostly horizontal (left/right) and the target wall was along the right side of the screen.
- Flash games. The BoMI had a library of flash games that the subjects could choose (e.g., Solitaire, Uno, or Arkanoid).

In the first block (familiarization block), the subjects began practicing and became acquainted with the BoMI. From session 5 the PT, thanks to a graphical user interface, introduced the modifications intended to encourage the cSCI subjects to recruit movement combinations that were more difficult to execute. The modifications consisted of:

(i). changing the contributions that each sensor gave to the movement of the cursor, by modifying the elements of the matrix H through the multiplication with the matrix D. D is a 2 × 8 matrix whose first row $d_{1}=\left[\begin{array}{ccc} d_{1,1} & \ldots & d_{1,8} \end{array}\right]$ contains the contribution of each sensors’ channel to the horizontal cursor movement, and the second row $d_{2}=\left[\begin{array}{ccc} d_{2,1} & \ldots & d_{2,8} \end{array}\right]$ the contribution to the vertical movement. All its elements were initialized to 1, and to change, for example, the contribution of all sensor channels to the horizontal movement it was sufficient to set the coefficients of $d_{1}$ >1.
(ii). changing the IMU signals by multiplying one or more IMUs by the gains contained in the matrix S, an 8 × 8 diagonal matrix. All the elements $s_{i}$ were initialized to 1, if then $s_{i}$ was set to be >1 the correspondent body signal $q_{i}$ increased.

These operations are already described in [29,31], are expressed as:

$$H_{change}=H\circ \left(D\cdot S\right)$$

where D is 2 × 8, S 8 × 8 and ∘ the Hadamard product that operates a pairwise multiplication between the elements of the two matrices. The GUI, through few textboxes and written indications, was helping the PTs to insert the numbers in order to operate all the necessary modifications. The modifications were applied before starting session 5, session 8, and session 10. This was done to gradually increase the difficulty of the exercise, keeping the subjects motivated and engaged in the training.

For all subjects, the goal was to shift the control toward more distal regions, e.g., from the upper- to the forearms, and if needed to promote symmetry in the use of right and left body sides.

To evaluate the effectiveness of training with the BoMI, the upper limb mobility was evaluated with clinical tests (see Section 2.4) and instrumental tests (see Section 2.5) at different time points: before BoMI treatment (T0), at the end of the BoMI treatment (T1) and 3 months after the end of the treatment (T2).

### 2.2. Subjects

We retrospectively analyzed the data of the cSCI subjects who, within a period of one year, underwent a training with the BoMI focused on distal upper limbs rehabilitation at the Santa Corona Hospital, in Pietra Ligure, Italy.

The inclusion criteria were: complete injuries at the C3–6 cervical level (American Spinal Injury Association, ASIA, grade A) or incomplete injuries in the cervical cord (ASIA B and C). They must be medically stable, able to see in adequate light, able to perform some shoulder and arm movements, and able to follow simple instructions. Five subjects matched these criteria (see Table 1 for diagnostic and demographic information). All data were collected as part of routine diagnosis and treatment. The ASIA grade reported in Table 1 was the result of the evaluation performed at the time of recruitment (1 week before starting the BoMI training). cSCI subjects were treated according to the national guidelines and to the ethical standards of the 1964 Declaration of Helsinki and signed an informed consent to the analysis of their data for research purposes. cSCI subjects’ data were compared with that of five unimpaired age and gender-matched adults with no history of neurological or muscular disorders. The study, the informed consent, and the publication of the results were approved by the local ethical committee (Comitato Etico Regione Liguria N.92366).

### 2.3. Data Analysis

#### 2.3.1. Learning Metrics for BoMI Training

To investigate if the subjects became skilled at controlling the cursor, we focused our analysis on the center-out movements only in the Reaching and the Pong tasks performed during the first and last session of the familiarization phase, the sessions before and after each interface modification and the session at the end of the training. For the Reaching task we computed the following metrics:

- Movement Time, time elapsed as the cursor reaches a target since it left the starting position;
- Linearity Index, length of the cursor trajectory to the external target normalized by the distance between start and end points. A linearity index equal to 1 means that the cursor moved along a straight line;
- Number of peaks in the velocity profile. We considered every peak larger than 15% of the maximum speed of each trajectory [33]. This is a measure of smoothness.

Those are simple metrics commonly used to evaluate controllability and movement quality since they allow describing both spatial and temporal performance as well as smoothness of the controlled effector (cursor). In the Pong game, we calculated the hit rate as the number of hits divided by the duration of the pong session (2.5 min).

#### 2.3.2. Symmetry and Distal Body Recruitment Indices in Body-Space and Task-Space While Using the BoMI

To analyze the symmetry and distality of control, we isolated the contribution of the Left L and right R side of the body and the Proximal upper arm P and the Distal forearm D to the cursor movement. The IMU signals were partitioned into 4 components, depending on which side of the body (right/left, R/L) and which portion of the arm (proximal/distal, P/D) they referred to. Therefore, we rewrite q as the sum of 4 vectors:

(2) $$q=q_{LD}+q_{LP}+q_{RP}+q_{RD}$$

where each term is an 8-dimensional vector with only two non-zero elements obtained from the corresponding IMU (Figure 1A: LF = IMU1, LU = IMU2, RU = IMU3, RF = IMU4). Substituting this expression in Equation (1), we determine how each side and each part of the body contributed to the total movement of the cursor:

(3) $$p=H\cdot \left(q_{LD}+q_{LP}+q_{RP}+q_{RD}\right)=p_{LD}+p_{LP}+p_{RP}+p_{RD}=p_{L}+p_{R}$$

Then, in the task space, we can compute the 2D trajectory derived only considering the signals of the sensors placed on the right body side (T_R_) or on the left side (T_L_). Defining PL as the operator that computes the path length of a trajectory, we can define the cursor contribution of the right body parts $c_{R}$ and of the left body parts $c_{L}$ as:

(4) $$\frac{PL\left(T_{R}\right)}{PL\left(T_{R}\right)+PL\left(T_{L}\right)}+\frac{PL\left(T_{L}\right)}{PL\left(T_{R}\right)+PL\left(T_{L}\right)}=c_{R}+c_{L}=1$$

We therefore calculated the symmetry index $c_{sym}$ in order to evaluate if the subject is using left and right body sides in a similar way as

(5) $$c_{sym}=\left(1-\left|c_{L}-c_{R}\right|\right)\cdot 100$$

if $c_{sym}\sim 100$ there is a symmetric condition in the cursor control. If $c_{sym}\to 0$ one of the sides is being used almost exclusively. In the same way, we computed the 2D trajectory derived considering the signals of the sensors placed on proximal portions of the arms, upper arms (T_P_) or on distal portions, the forearms (T_D_) and calculated their contributions to cursor movement, respectively $c_{P}$ and $c_{D}$ as

(6) $$\frac{PL\left(T_{P}\right)}{PL\left(T_{P}\right)+PL\left(T_{D}\right)}+\frac{PL\left(T_{D}\right)}{PL\left(T_{P}\right)+PL\left(T_{D}\right)}=c_{P}+c_{D}=1$$

To determine whether subjects used more distal body parts, we considered the index $c_{distal}$:

(7) $$c_{distal}=c_{D}\cdot 100$$

if $c_{distal}$ is increasing the subject was using more the distal part of the body.

The same approach was used in the body space to compute the relative contribution of each body side ($b_{L}$ and $b_{R}$) and district ($b_{P}$ and $b_{D}$) from the standard deviations (std) of the 2 channels (pitch and roll) of each IMU:

(8) $$\frac{std_{pitch1}+std_{roll1}}{std_{tot}}+\frac{std_{pitch2}+std_{roll2}}{std_{tot}}+\frac{std_{pitch3}+std_{roll3}}{std_{tot}}+\frac{std_{pitch4}+std_{roll4}}{std_{tot}}\equiv b_{LD}+b_{LP}+b_{RP}+b_{RD}=b_{L}+b_{R}=1$$

with $std_{tot}=std_{pitch1}+std_{roll1}+std_{pitch2}+std_{roll2}+std_{pitch3}+std_{roll3}+std_{pitch4}+std_{roll4}$ Similar to Equations (5) and (7), we defined $b_{sym}$ and $b_{distal}$ as

(9) $$b_{sym}=\left(1-\left|b_{L}-b_{R}\right|\right)\cdot 100$$

(10) $$b_{distal}=\left(b_{LD}+b_{RD}\right)\cdot 100$$

Also, in this case, with high symmetry we will have $b_{sym}\sim 100\%$ and with a greater use of the distal body parts $b_{distal}\sim 100\%$. Differently from the indicators computed from the trajectories in the task space, the ones extracted directly from the IMUs output are not influenced by the BoMI mapping. Therefore, they account for the actual body movements. We should consider that the movements of the upper arm influence both readouts of the distal and proximal sensors. So, for example, in the arm kinematic chain a movement of the upper arm results also in a movement of the forearm. Thus, decoupled distal movement due to the elbow joint, that are the main target of the BoMI-based exercises proposed in this work, are related to symmetry values above the 50%.

We computed these indicators during two sessions: (a) session 4, the last session of the familiarization phase, and (b) session 15, the last session of the training phase.

### 2.4. Clinical Evaluations

According to prevalent clinical practice we decided to use the Manual Muscle Test (MMT) [34], performed by expert clinicians blind to the subjects’ training, to assess upper body strength. In particular, we focused on three upper body regions: scapulae, shoulders, and arms. See Table S1, for details on the tested movements. Each movement was evaluated with a number from 0 (no movement) to 5 (normal movement). The maximum achievable score for the scapula is 15, for the shoulder is 30, and for the arm is 10.

To assess upper body mobility, we measured the Range of Motion (ROM) of the shoulders and arms in all the possible directions using a goniometer, see Table S2 for more information. Since cSCI subjects were tested while sitting in their wheelchair, we did not include shoulder adduction and shoulder extension measures due to substantial range of motion limitations while being in this position.

### 2.5. Instrumented Evaluation—Stabilization Task

**Figure 2:**
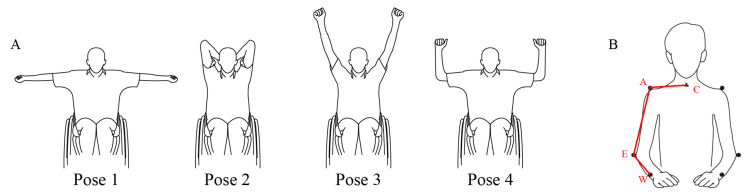
(**A**) The poses of the arm *stabilization task* from the Van Lieshout Test (VLT) manual that were evaluated in this study, from the easiest (Pose 1) to the most difficult one (Pose 4). (**B**) Markers placement on the anatomical landmarks of acromion (A), elbow (E), wrist (W) and C7 (C) used in the kinematic analysis. The marker on C7 is displayed in the figure, despite the frontal view, to simplify the visualization.

We also evaluated using an instrumented test the BoMI training’ effects on movement kinematic before (T0), at the end (T1), and 3 months after the end of training (T2). There were some missing data for the cSCI population: subject SCI2 did not perform the instrumented evaluation at T1, while subject SCI4 did not perform the instrumented evaluation at T2. The instrumented evaluation was selected from those presented in the Van Lieshout Test (VLT) Manual [35]. The reason behind the choice of such task was that, differently from the tests usually adopted in the clinical practice, it involved both arms at the same time and required multi-joint coordinated movements in three-dimensional space. The clinical version of the VLT consists of 19 items divided into 5 areas of interest: arm ability to transfer the body, arm positioning and stabilizing, hand opening and closing, grasping and releasing, and manipulating. In particular, we chose the second area of interest, namely the stabilization, which is composed of a set of five poses (see Figure 2A) that assess the ability to freely stabilize the arms in space against gravity during five seconds for each pose. Every subject performed a session composed of six repetitions for each pose for a total of 30 movements. The poses were characterized by an increasing level of difficulty, from pose 1 to pose 5.

In the first pose, the arms were positioned horizontally in the lateral direction, and the elbows were completely extended while the thumbs pointed posteriorly. The second pose consisted of setting the elbow to point upward with hands that touched the neck and vertical forearms near the head. Pose 3 instead required to extend the arms over the head fully; they could be positioned slightly apart and not necessarily exactly vertically with the elbow fully extended. In the fourth pose, the arms were set horizontally in the lateral direction, with elbows flexed of 90 degrees in outward rotation. Finally, in pose 5, the arms were stabilized horizontally in the lateral direction with elbows fully extended, and the thumbs point downward. In this study, we excluded pose five from the task sequence because none of the subjects could perform this pose due to the level of their injury. For each pose, we could distinguish an execution phase, consisting in the time elapsing from the instant the subject left the starting position to the reach of the desired pose, and a holding phase, consisting in the 5 s during which the subject has to maintain the pose.

#### 2.5.1. Kinematic Recordings

During the execution of the stabilization task, the kinematics for the upper body was acquired. We used a motion capture system (SMART DX, BTS Bioengineering, Italy) with 8 infrared cameras and 2 video cameras (Vixta). The global reference of the system was located with the *X-axis* along the sagittal plane of the subject, the *Y-axis* along the vertical direction and the *Z-axis* along the frontal plane. To reduce the variability of the marker placement among sessions, the same person was in charge of placing markers. We located 13 markers (15 mm diameter) on anatomical landmarks according to the Davis protocol [36]: head, 7th cervical vertebra, right scapula, left scapula, sternum, right acromion, left acromion, right elbow, left elbow, right wrist, left wrist, right metacarpus, and left metacarpus. The kinematic data were recorded at a sampling frequency of 100 Hz.

#### 2.5.2. Visual Analysis

A physical therapist evaluated the performance in the VLT by assigning a score to each pose’s repetition by visual inspection of the recorded task. Then, for every pose, the scores obtained in each repetition were summed and normalized by the maximum value that could have been reached to have a range of values from 0 to 1.

#### 2.5.3. Kinematic Analysis

The movements were sampled at 100 Hz and smoothed by a 4th order Savitzky-Golay filter (cutoff: ~15 Hz), which was also used to estimate the subsequent time derivatives of the trajectory. For each pose, movement onset was defined as the first time instant when the speed of the marker placed on the wrist exceeded 10% of the peak speed in that phase. The movement end was defined as the last time sample the speed reached the minimum value. This allowed the inclusion of possible movement adjustments. Indicating as C, W, E, and A the locations of the markers placed respectively on C7, wrist, elbow and acromion (Figure 2B) we also extracted the following parameters for right and left body side:

- Elbow Angle (EA). The angle $\hat{WEA\;}$ formed by the segments WE and EA;
- Shoulder Angle on Frontal plane (SAF). The angle $\hat{CAE\;}$ formed by the segments CA and AE projected on the frontal plane;
- Shoulder Angle on Sagittal/Transverse plane (SAST). The angle $\hat{CAE\;}$ projected on the sagittal plane (for pose 2 and pose 3) or on the transverse plane (for pose 1 and pose 4);

It should be noted that this kinematic analysis is not complete in a geometrical and physiological sense as it does not consider for example rotations of the forearm along the elbow-wrist axis. Kinematic Symmetry ($K_{sym}$). This metric evaluates if the subject used left and right body side in a symmetric way while holding the different poses. It was computed by averaging together the symmetry indicator extracted from the three previously defined parameters:

(11) $$K_{sym}=\frac{EA_{sym}+SAF_{sym}+SAST_{sym}}{3}$$

where each kinematic measure of symmetry ($EA_{sym}$, $SAF_{sym\;}$ and $SAST_{sym}$) is defined as the ratio of the indicator computed from data of the right side of the body to the indicator computed from data on the left.

### 2.6. Statistical Analysis

The small sample size of our population did not allow for appropriate full statistical analysis of the data to assess the significance of the changes in the performance metrics during the BoMI training and during the instrumented evaluation. However, consistencies were tested using a Wilcoxon signed-rank test with 5 matched pairs [37,38] (Matlab function signrank). We acknowledge that because the small sample size the significance is debatable, but we still report the *p*-values in order to give an idea of the common trend, if any, of the population. The level of significance has been set as follows for the signed-rank test: p*** = 0.03 if all five differences were in the same directions, p** = 0.06 if 4 out of 5 differences were in the same direction with the non-conforming difference being the smallest in magnitude, p* = 0.09 if 4 out of 5 differences were in the same direction, with the non-conforming difference between the second smallest in magnitude.

A Wilcoxon signed-rank test was run on the learning metrics, regarding the Reaching task: we compared the session 1 of reaching 1 with the last of reaching 2. We ran the Wilcoxon signed-rank test on the metrics describing the reorganization of body movement, symmetry ($c_{sym}$ and $b_{sym}$) and body parts recruitment ($c_{distal}$ and $b_{distal}$) on session 4 and session 15.

We also compared the MMT and ROM results obtained during T0 and T1 to check for changes due to the BoMI treatment and the ones obtained during T1 and T2 to evaluate if eventual positive changes were still maintained after 3 months.

For the analysis of the instrumented evaluation (stabilization task), for each parameter, we obtained a single indicator as the average of the four poses, and we compared the performance of the 5 subjects pre and post treatment (T0 and T1) to check for changes due to BoMI training and we compared post-treatment and follow-up (T1 and T2) to verify if the performance were maintained after three months. For the kinematic parameters (*EA*, *SAF* and *SAST*), we removed the “normality” baseline (control group performance) from the cSCI population before running statistical tests.

## 3. Results

### 3.1. SCI Subjects Learned to Use the Interface

**Figure 3:**
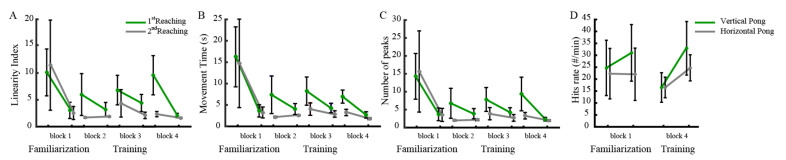
Performance metrics. Reaching tasks: linearity index (**A**), movement time (**B**) and number of peaks in the velocity profile (**C**). First and last sessions of the familiarization phase, first and last sessions after the 1st, 2nd and 3rd map modification of the training phase. Pong hits rate during vertical and horizontal pong (**D**). For the Pong tasks we are reporting only values at the beginning (respectively session 3 for vertical pong and session 4 for the horizontal pong) and end of the familiarization and at the beginning and end of the last change of the interface. We reported the mean values and standard error of the subjects.

All cSCI subjects practiced for a long time and quickly learned to proficiently operate the interface also in this experiment, where the sensors were placed on more distal parts than in previous studies [15,28], Figure 3. During the familiarization phase (block 1), they improved the cursor control skills in reaching tasks, becoming faster and making straighter and smoother trajectories (Figure 3A–C).

As expected, after modifying the BoMI map to encourage the use of more distal segments and a similar involvement of right and left sides (beginning of blocks 2–4), the performance worsened. When the BoMI induced the subjects to generate more forearm movements, the cursor became more jittery as showed by the increase of our jerk index (the number of peaks in the velocity profile, Figure 3C). However, at the end of training (end of block 4), the reaching metrics were comparable to the metrics at the end of the familiarization phase (end of block 1). Globally, all subjects improved their performance from beginning to end of the BoMI training in terms of the linearity index (*p* = 0.0312), the movement time (*p* = 0.0312), and the number of peaks (*p* = 0.0312). Instead, in both pong games, three subjects increased the hits rate from the beginning to the end of the training, while one maintained constant performance and one showed a slight decrease (*p* = 0.2188, vertical pong, *p* = 0.312 horizontal pong).

### 3.2. Increased Recruitment of Distal Regions of the Arms

**Figure 4:**
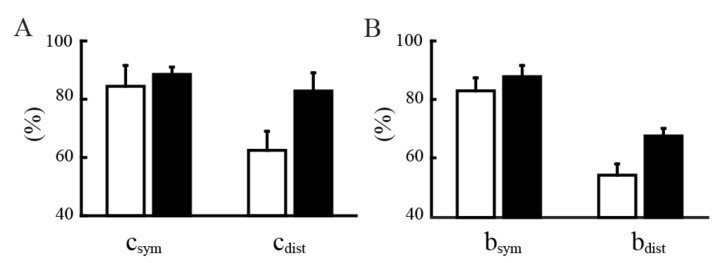
Symmetry and distality indices for body contribution to cursor movement (**A**) and for body mobility (**B**) at the end of the familiarization phase (white bars) and end of training (black bars). The indices are calculated for representing the symmetry between right and left upper body ($c_{sym}$ and $b_{sym}$) and for representing the usage of more distal body parts ($c_{dist}$ and $b_{dist}$).

The training mostly focused on increasing the use of more distal parts and the symmetry of the right and left sides. The data presented in Figure 4 show that training indeed influenced the recruitment of the upper limbs’ distal parts. The subjects at the end of the familiarization contributed to the cursor control with high symmetry (above 80%, with 100% indicating perfect symmetry), but with different use of the upper arms and forearms (Figure 4A), the same trend when looking at the total mobility of the upper body (Figure 4B). In this case the goal was to encourage the use of the forearms and this objective was reached at the end of the training with the BoMI.

All the subjects increased, in general, their mobility, maintaining symmetrical body recruitment; in fact $c_{sym}$ and $b_{sym}$ did not show differences at the end of the training with respect to the end of familiarization (respectively *p* = 0.4062 and *p* = 0.1562). Noticeably, when we compared the usage of the body parts that they had more difficulty controlling, in this case the forearms, all the users increased the percentage of the forearm recruitment contributing to cursor control ($c_{dist}$) at the end of the training, going from 61.63 ± 7.38% on session 4 to 82.77 ± 6.28% on the last session (*p* = 0.0312). Similarly, when looking at the mobility of the forearm, $b_{dist}$ went from 54.46 ± 3.29% to 61.94 ± 2.99% (*p* = 0.0312). Note that values above the 50% indicate, as explained in the methods, a contribution of the distal (elbow) movements decupled from the proximal (shoulder) joint motions.

### 3.3. Improvements in the Clinical Evaluation Tests

**Figure 5:**
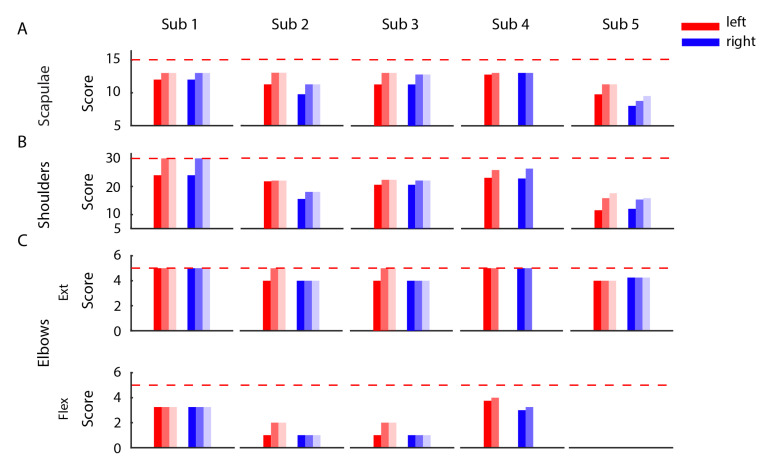
Manual muscle test. Each row presents the results of the MMT for the left (shades of red) and right (shades of blue) body parts of each of the 5 subjects recruited in the study performed before (dark shade), at end (medium shade) and 3 months after the end of the training (light shade). The scores are divided by body districts (rows) and the dashed horizontal red lines correspond to the maximum score that could be assigned to each district.

**Figure 6:**
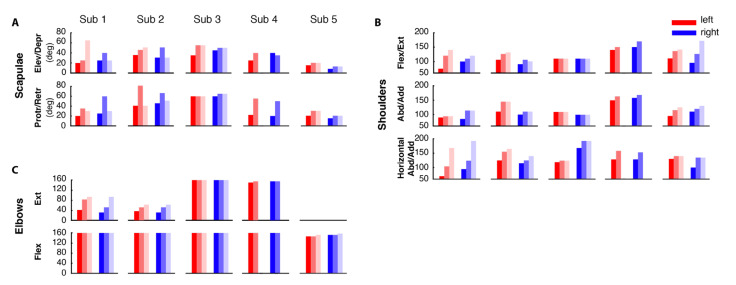
Range of motion. Results of the Range of Motion (ROM) for each subject before (dark shade), at the end (medium shade) and 3 months after the end of the training (light shade) for the left (shades of red) and right (shades of blue) body parts. The results are presented divided by upper-body districts: scapulae (**A**), shoulders (**B**) and elbows (**C**).

The clinical evaluation tests, MMT (Figure 5) and ROM (Figure 6) performed before, immediately after, and three months after the end of the training showed a positive effect of the BoMI in SCI rehabilitation for all subjects. At training completion, all subjects had an increase of muscle strength (*p* = 0.0312 for the left body parts and *p* = 0.0312 for the right of the entire population). The improvement was maintained at the follow-up (comparing the results for the left side and for the right side at the end of the training with the ones at the T2 we obtained no changes *p* = 0.5). The same trend was also evident in the outcomes of the ROM. All the subjects exhibited an increase in the range of movements of shoulders, arms and forearms (*p* < 0.001); the only measure that did not change was elbow flexion because all subjects had a complete elbow flexion since the beginning.

There was no noticeable change between the end of training and the follow-up for the right side of the body (*p* = 0.2318) while a change was still present for the left side (*p* = 0.0139).

### 3.4. Kinematics for the Stabilization Task

**Figure 7:**
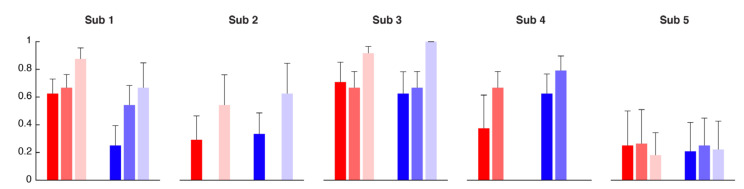
Normalized scores assigned to each body side (right in blue and left in red) averaged across the four poses performed by each subject (columns). The evaluation was performed before the BoMI treatment (T0, dark shades), at the end of the BoMI treatment (T1, medium shades) and three months after (T2, light shades).

The subjects’ kinematic during the arm stabilization task was assessed in two ways: by a visual inspection of the video recordings and subsequent scoring of the performance, and by extracting significant parameters from the markers placed on the upper limbs. Figure 7 reports the mean scores given to all the poses performed by each subject at the different time points (T0, T1 and T2).

The entire SCI population showed an improvement in all the poses between T0 and T1, especially for the right side of the upper limb (*p* = 0.0312). Pose 1 was the one that all the subjects were able to perform almost from the beginning; for the other poses, there was a clear trend of improvement from T0 and T1. The improvement was also maintained at T2. Subject 5, being the one with the highest level of lesion and so more impaired, was the subject that obtained the lowest scores being not able to perform the pose 2 and 4 in none of the evaluation sessions and the pose 3 only at T1 and T2.

**Figure 8:**
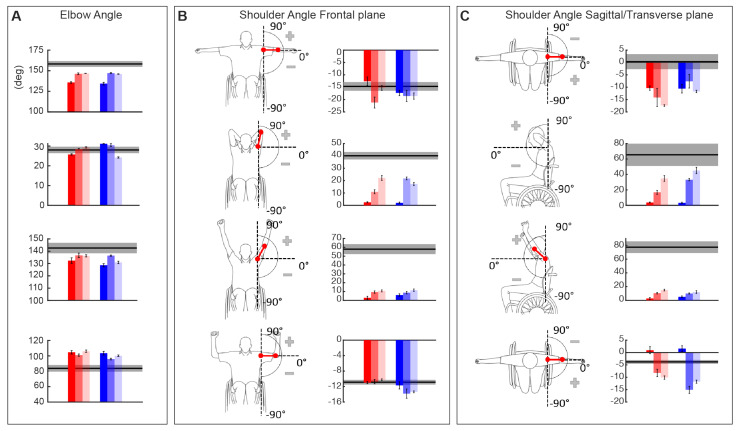
Kinematic parameters of the stabilization task for an example subject, SCI1. In each panel each row indicates the parameters relative to pose 1, pose 2, pose 3 and pose 4. In the shades of red the parameters extracted from the left body parts while in the shades of blue the one from the right body parts at T0 (dark shades), T1 (medium shades) and T2 (light shades). The grey area in each graph represents mean and standard error of each parameter for the control subjects (mean ± SE). (**A**) Elbow Angle—EA. (**B**) On the left a schematic description, for each pose, of the computed angle and on the right the results of the Shoulder Angle on the Frontal plane—SAF. (**C**) Schematic description, for each pose, of the computed angle on the left side and Shoulder Angle on the Sagittal/Transverse plane -SAST- graphs on the right.

Figure 8 depicts the trend of all the three kinematic parameters EA, SAF, and SAST computed for a representative subject (SCI1), respectively, to evaluate distal movements (EA) and proximal movements (SAF and SAST) for both left and right body side. This subject showed a global improvement in EA, while the other two kinematic parameters improved only for pose 2 and 3. The individual results for the other subject are similar and reported in the Supplementary Materials.

**Figure 9:**
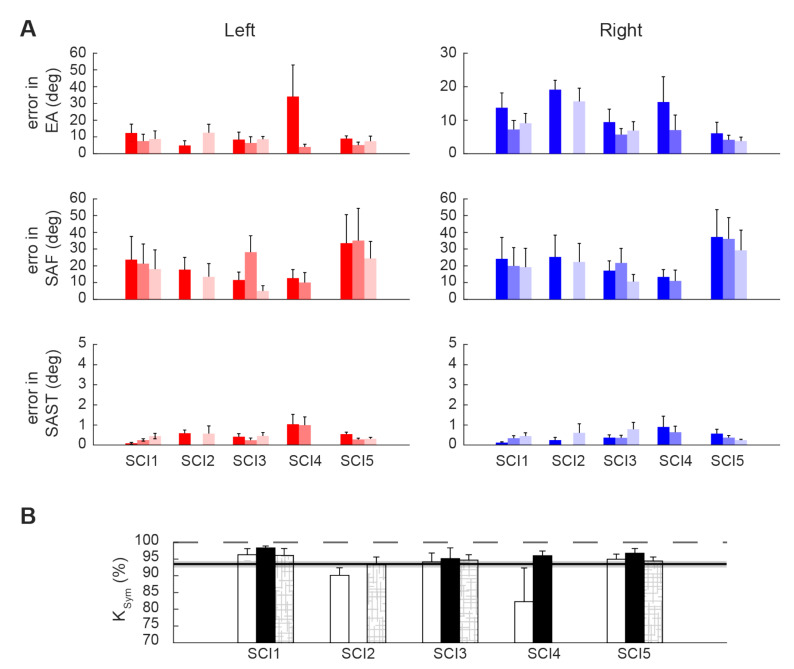
(**A**) Kinematic parameters of the stabilization task for the cSCI population normalized with respect to the control population. Elbow Angle, (EA, first row), Shoulder Angle on Frontal plane (SAF, second row) and on Sagittal/Transverse plane (SAST, third row) were averaged across poses for each subject, mean and standard error are reported in the figure. Shades of red represent the parameters extracted from the left body parts while in the shades of blue the ones from the right body parts at T0 (dark shades), T1 (medium shades) and T2 (light shades). (**B**) Overall kinematic symmetry parameter, $K_{sym}$, computed for each cSCI subject at T0 (white bars), T1 (black bars) and T2 (patterned bars).

The kinematic performance of the entire cSCI population is reported in Figure 9 where *EA*, *SAF,* and *SAST* metrics are displayed as distance from the control group values. Thus, the metrics close to zero indicated performance similar to the healthy ones, highlighting that subjects were able to correctly achieve the required postures. We found that after treatment (T0-T1), for all the poses, four out of five SCI subjects reported an improvement for the *EA* metrics (p** = 0.06 for both left and right body parts), and this improvement was maintained in the follow-up evaluation (T1-T2: *p* = 0.875 for the right side and *p* = 1 for the left side). This result supports the findings previously described after the BoMI training, and it is an additional proof that improving distal body parts movements with the BoMI training was actually achieved. Conversely, the two kinematic parameters related to the proximal movements, *SAF* and *SAST*, did not reveal an overall improvement for the cSCI subjects in both T0-T1 comparison (*SAF*: *p* = 0.43 for right body parts, *p* = 0.56 for left body parts; *SAST*: *p* = 0.3125 for right, *p* = 0.1857 for left) and T1-T2 comparison (*SAF* metrics: *p* = 0.12 for both sides; *SAST*: *p* = 0.75 for right side, *p* = 1 for left side). As for the *Kinematic Symmetry* ($K_{sym}$), this parameter (Figure 9) improved between T0 and T1 for all the poses for 4 subjects out of 5 (p** = 0.0625). No differences were evidenced between T1 and T2 (*p* = 1). Therefore, the performance achieved at the end of the training was maintained at follow-up.

## 4. Discussion

The BoMI presented in this study is a rehabilitative tool tested in a clinical environment that provides therapists with a simple technology with a high potential to help the training and recovery of upper limb movements of acute cervical SCI subjects. In this study, we described and quantified the efficacy of its use for distal movement skills recovery. Concurrently, we described the effects of the BoMI training not only based on clinical scales but also on an instrumented test that engaged the subjects in bilateral arms movements towards poses of increasing difficulties. In previous studies, a similar BoMI was tested, but these either focused on proving the BoMI as an assistive tool for operating a computer or a virtual and powered wheelchair [23,28] or they demonstrated the capability of the BoMI to operate both as an assistive and as a rehabilitative tool, always working with chronic cSCI subjects and with sensors placed on proximal regions, i.e., shoulders and upper arms [15,29]. Here, we analyzed data of 5 cSCI subjects, in their acute phase with the only exception of SCI 3 that went through a period of training with the BoMI as part of their inpatient rehabilitation routine. Sensors were also moved in distal positions, being all the cSCI subjects able to exert some kind of control on their forearms and not only on their arms. Despite these differences with previous works, all subjects while playing reaching and pong games had a final behavior and performance comparable to the one of the study where the sensors were involving upper arms [15], no longer only shoulder movements like in [28]. Specifically, in studies where the control involved more distal movements, either shoulders and arms or arms and forearms movements, the initial performance was worse than when the control involved only shoulder movements. However, despite this sensors’ location, after a short period of four sessions, all the subjects became proficient in the control, with movement time and smoothness performance similar to those observed in previous studies, including the works based on shoulder movements.

From the BoMI data, we also extracted indicators regarding body movements and body contributions to cursor control, that allowed the PTs to modify the interface to reach the individual rehabilitation objectives included in the recovery plan of each cSCI subject. All of them at the end of the familiarization phase were using the BoMI with similar recruitment of right and left upper body, therefore for everyone, the main rehabilitative goal was to increment the movements of the forearms over the arms. Indeed, using the forearms for a cervical SCI subject is more challenging due to a reduced innervation of peripheral muscles because of the lesion location on the cervical tract of the spinal cord [1,39]. The BoMI parameters’ modifications succeeded in pushing the subjects to increase forearm movements, still maintaining a symmetrical body use. This was also confirmed by the results obtained from the instrumented evaluation. All the standard clinical tests, MMT and ROM, for all the cSCI subjects improved between pre and post assessments (T0 vs. T1). Also, the results of the stabilization task had the same trend both looking at the score provided by expert clinicians and at the kinematic data of the instrumented VLT. With training, all cSCI subjects improved and got closer to the posture assumed by the control subjects. The kinematic parameters that had the greatest improvement were the elbow angles, i.e., the main target of our new BoMI-based training. This finding confirms that this rehabilitation training aimed mainly to improve distal body parts’ functionality, fundamental for several daily life tasks, lasted at follow-up. The choice of using and instrumenting the VLT test, a test that has been proven to be valid, reliable and responsive [40,41], was motivated by the need for having a test that consisted of a bilateral task as support of the standard clinical tests, where each district is evaluated singularly. Moreover, we wanted to extract kinematic indicators that could help doctors and PTs to objectify progress during and after a rehabilitative program in a clinically meaningful way and to use these indicators together with standard MMT and ROM, which often lacks objectivity, to answer questions like: is this level of functioning a satisfying result in this phase of treatment? Is the current level of functioning the best that can be reached by the subject? 

The limitations of this work mainly regard the sample size of cSCI subjects and the lack of a control cSCI group. It is worth noting that since the cSCI subjects, except SCI 3, were in the acute phase, the improvements might be at least partially due to the spontaneous recovery happening during this early stage of the injury. A control cSCI group would have allowed us to decouple the effects of spontaneous recovery, traditional rehabilitation training, and the BoMI-based treatment. However, the enhanced improvement in the kinematic parameters targeted by the BoMI suggests a positive synergy between these factors, to further investigate in a future study. An additional limit of this study is in the low number of cSCI subjects recruited. Cervical SCI is a relatively rare condition. Further studies including larger cohorts of participants will be necessary to draw more robust general conclusions. Finally, two subjects missed one of the three evaluation sessions. This was due to a personal impediment to participate in that particular session that the experimenter could not anticipate. This is not an uncommon occurrence in SCI subjects, who may experience sudden and unexpected medical problems or complications in the acute phase.

## 5. Conclusions

This study validated the feasibility of using BoMI as a complementary tool for SCI rehabilitation in the clinical environment. The functional evaluation protocol proposed and tested resulted easy to apply and well tolerated by people with cervical SCI. This protocol and selected indicators augmented the standard clinical evaluations and permitted to quantify with mode details the improvement brought by the BoMI training, combined with the standard rehabilitation treatment.

## Figures and Tables

**Table 1:** cSCI subjects’ characteristics. The fourth column reports the level of lesion and the American Spinal Injury Association (ASIA) impairment scale [32], indicating how much sensory or motor function is preserved. Grade A: Complete. No sensory or motor function preserved. Grade B: Incomplete. Sensory function preserved but no motor functions. Grade C: Motor Incomplete (less than half of the key muscle functions below the neurological level have a muscle grade ≥ 3). Grade D: Motor Incomplete (at least half or more of key muscle functions below the neurological level have a muscle grade ≥ 3). Grade E: Normal motor and sensory functions.

Subjects	Gender	Age	Level of Injury	Time after Injury
SCI 1	Male	21	C5 ASIA A	4 months
SCI 2	Male	19	C5 ASIA B	6 months
SCI 3	Male	20	C6 ASIA C	6 years
SCI 4	Female	29	C5 ASIA A	6 months
SCI 5	Female	28	C5 ASIA A	3 months

## Data Availability

The data presented in this study are available on request from the corresponding author. The data are not publicly available due to patient privacy considerations (HIPPA).

## Supplementary Materials

The following are available online at https://www.mdpi.com/1424-8220/21/6/2243/s1, Figure S1, Kinematic parameters of the stabilization task for subject SCI2; Figure S2, Kinematic parameters of the stabilization task for subject SCI3; Figure S3, Kinematic parameters of the stabilization task for subject SCI4; Figure S4, Kinematic parameters of the stabilization task for subject SCI5; Table S1, Muscles tested with the manual muscle test (MMT); Table S2, Movements evaluated with the goniometer to extract the range of motion of the upper body in the frontal, sagittal and transverse plane.
