# Supplementary material for: Recovery of Distal Arm Movements in Spinal Cord Injured Patients with a Body-Machine Interface: A Proof-of-Concept Study
Source: Sensors (Basel). 2021 Mar 23;21(6):2243. doi: 10.3390/s21062243 (PMC8004832; doi:10.3390/s21062243)
Supplement: Supplementary file 1 [file sensors-21-02243-s001.pdf]

1 Supplementary material

Table S1. Muscles tested with the manual muscle test (MMT).

Body Part	Movement - Muscle
Scapula	Elevation – Upper Trapezius<br>Adduction – Rhomboids<br>Abduction – Serratus Anterior
Shoulder	Flexion – Anterior Deltoid<br>Extension – Posterior Deltoid<br>Abduction – Medial Deltoid<br>Adduction –Pectoralis Major<br>Horizontal Adduction – Pectoralis Major/Clavicula<br>Horizontal Abduction – Posterior Deltoid
Elbow	Flexion – Biceps Brachii<br>Extension – Triceps Brachii

Table S2. Movements evaluated with the goniometer to extract the range of motion of the upper body in the frontal, sagittal and transverse plane.

Plane	Movement
Frontal	Shoulder Elevation<br>Shoulder Depression<br>Shoulder Abduction
Sagittal	Shoulder Protraction<br>Shoulder Retraction<br>Shoulder Flexion
Transverse	Shoulder Horizontal Adduction<br>Shoulder Horizontal Abduction<br>Elbow Flexion<br>Elbow Extension

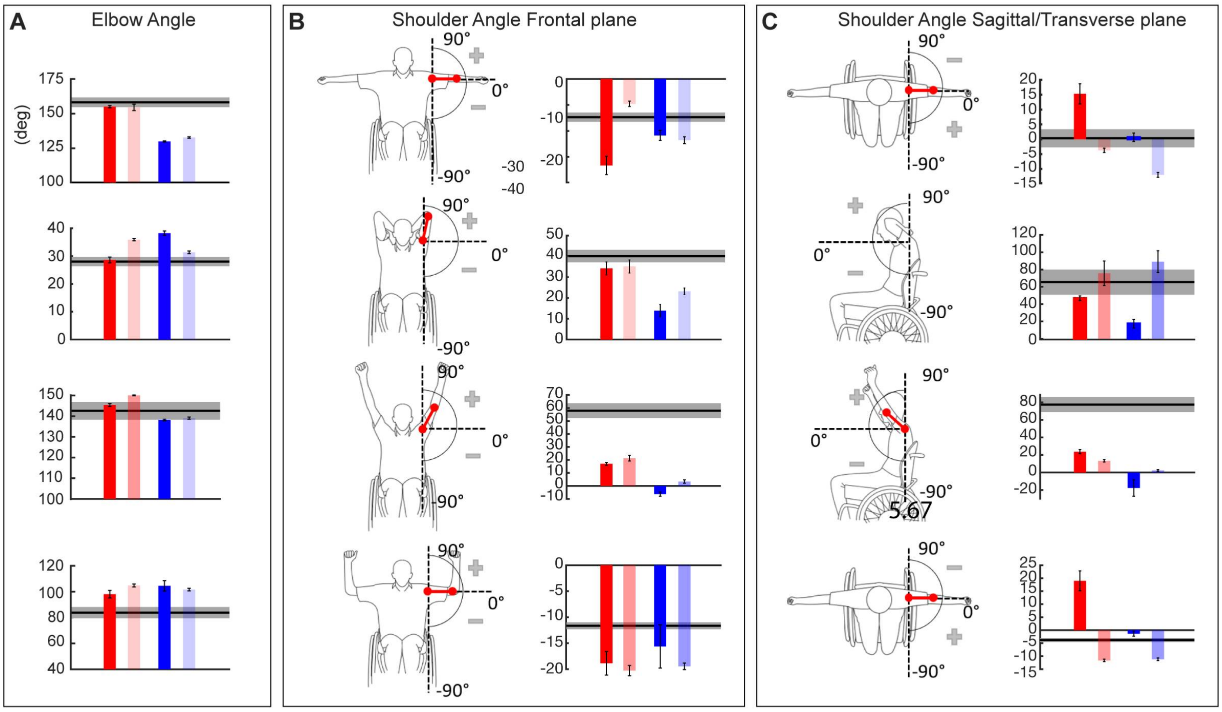

Figure S1: Kinematic parameters of the stabilization task for subject SCI2. The rows of each panel indicate the parameters relative to pose 1, pose 2, pose 3 and pose 4. In the shades of red the parameters extracted from the left body parts while in the shades of blue the one from the right body parts at T0 (dark shades), T1 (medium shades) and T2 (light shades). The grey area represents mean and standard error of each parameter for the control subjects. (**A**) Elbow Angle. (**B**) Shoulder Angle on the Frontal plane. (**C**) Shoulder Angle on the Sagittal/Transverse plane.

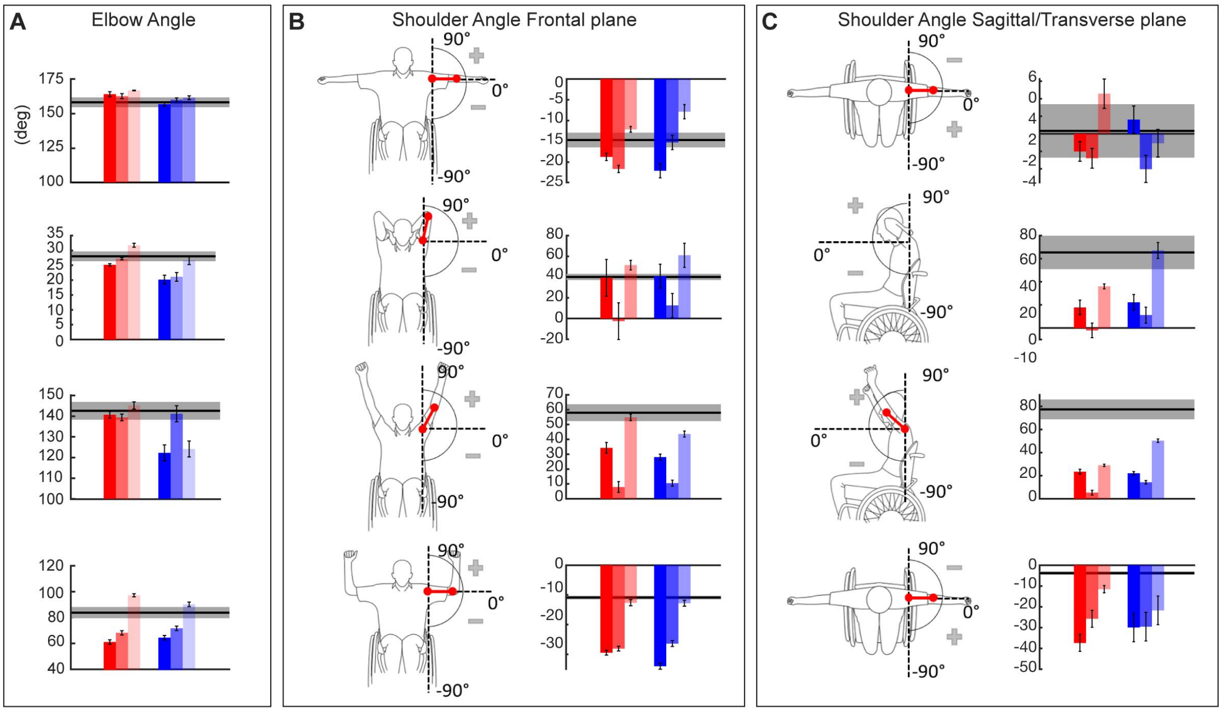

Figure S2: Kinematic parameters of the stabilization task for subject SCI3. The rows of each panel indicate the parameters relative to pose 1, pose 2, pose 3 and pose 4. In the shades of red the parameters extracted from the left body parts while in the shades of blue the one from the right body parts at T0 (dark shades), T1 (medium shades) and T2 (light shades). The grey area represents mean and standard error of each parameter for the control subjects. (**A**) Elbow Angle. (**B**) Shoulder Angle on the Frontal plane. (**C**) Shoulder Angle on the Sagittal/Transverse plane.

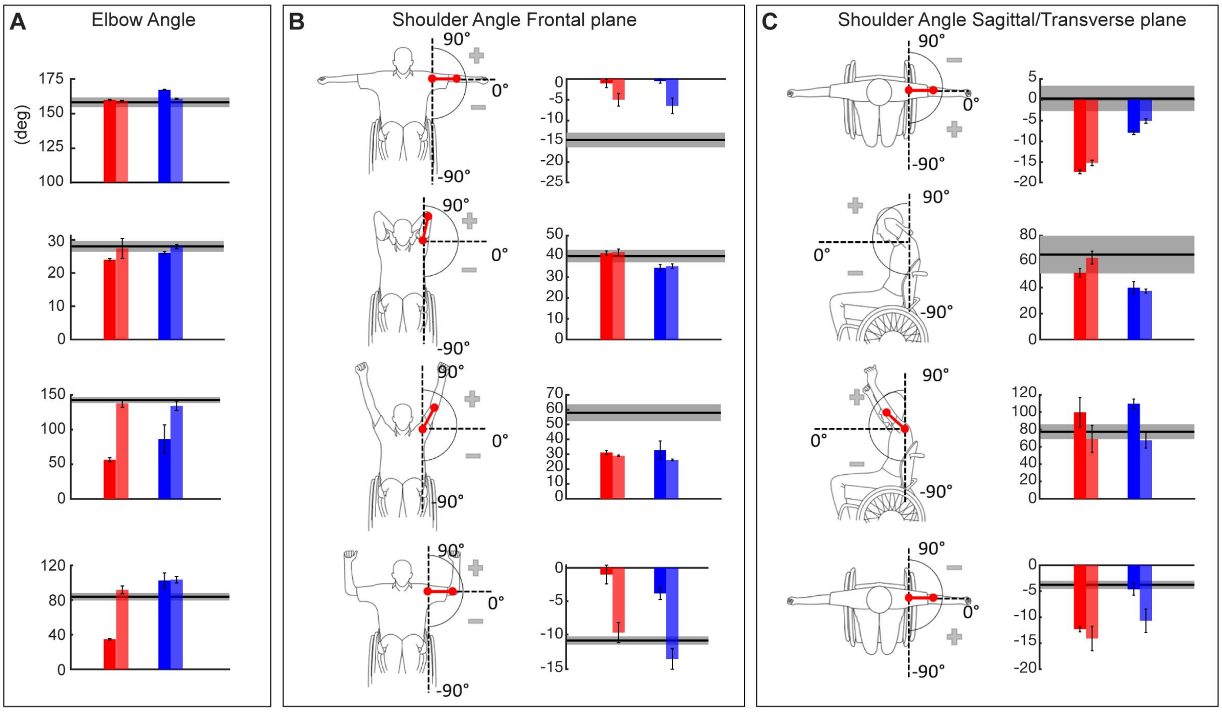

Figure S3: Kinematic parameters of the stabilization task for subject SCI4. The rows of each panel indicate the parameters relative to pose 1, pose 2, pose 3 and pose 4. In the shades of red the parameters extracted from the left body parts while in the shades of blue the one from the right body parts at T0 (dark shades), T1 (medium shades) and T2 (light shades). The grey area represents mean and standard error of each parameter for the control subjects. (**A**) Elbow Angle. (**B**) Shoulder Angle on the Frontal plane. (**C**) Shoulder Angle on the Sagittal/Transverse plane.

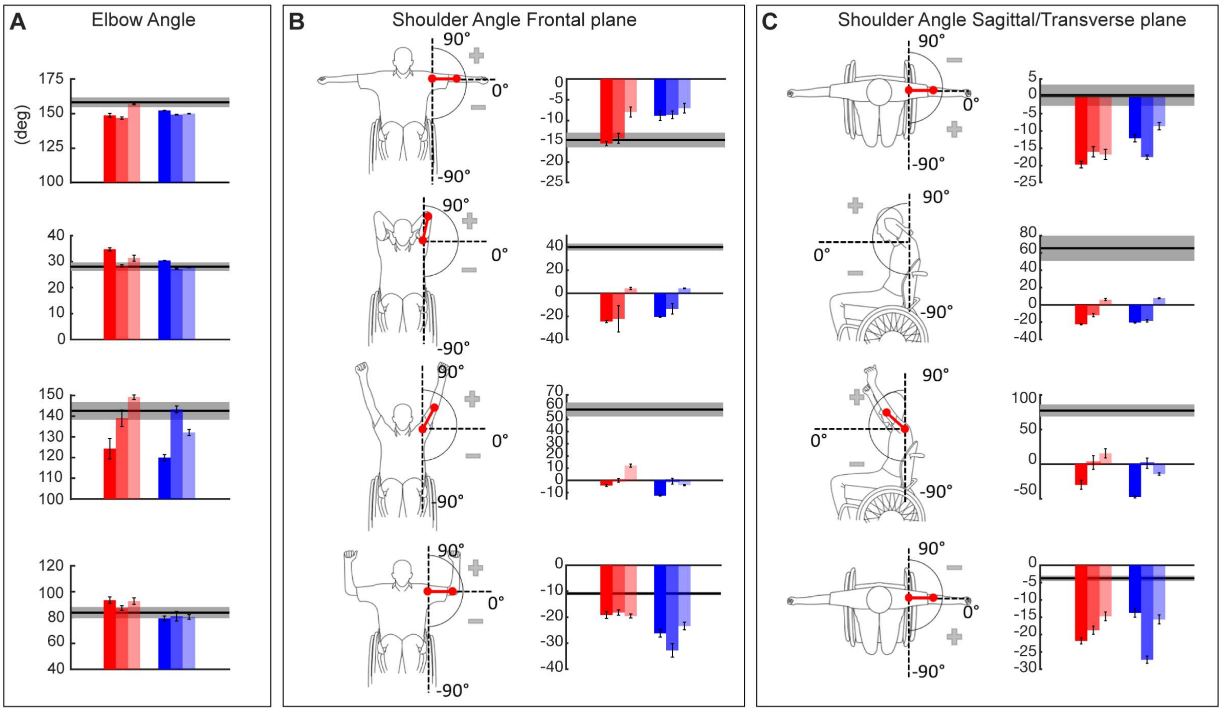

Figure S4: Kinematic parameters of the stabilization task for subject SCI5. The rows of each panel indicate the parameters relative to pose 1, pose 2, pose 3 and pose 4. In the shades of red the parameters extracted from the left body parts while in the shades of blue the one from the right body parts at T0 (dark shades), T1 (medium shades) and T2 (light shades). The grey area represents mean and standard error of each parameter for the control subjects. (**A**) Elbow Angle. (**B**) Shoulder Angle on the Frontal plane. (**C**) Shoulder Angle on the Sagittal/Transverse plane.
